# Isoperistaltic Versus Antiperistaltic Side-to-Side Ileocolic Anastomoses After Right Colectomies: A Systematic Review and Meta-Analysis

**DOI:** 10.7759/cureus.85603

**Published:** 2025-06-09

**Authors:** Bernardo F Pompeu, Julia Hoici Brunini, Marília Piassi Velucci, Lucas Guedes, Gabriel Leal Barone, Beatriz D´Andrea Pigossi, Sérgio Mazzola Poli De Figueiredo, Fernanda Formiga

**Affiliations:** 1 General and Colorectal Surgery, University of São Caetano do Sul, São Paulo, BRA; 2 General Surgery, Hospital Heliópolis, São Paulo, BRA; 3 Medicine, Universidade Municipal São Caetano do Sul, São Caetano do Sul, BRA; 4 Medicine, University of São Caetano do Sul, São Paulo, BRA; 5 Abdominal Wall, Cleveland Clinic, Cleveland, USA; 6 Colorectal Surgery, Santa Casa de São Paulo, São Paulo, BRA; 7 Colorectal Surgery, Hospital Heliopolis, São Paulo, BRA

**Keywords:** antiperistaltic, colorectal surgery, ileocolic anastomosis, isoperistaltic, meta-analysis

## Abstract

After right colectomy, ileocolic anastomoses can be configured as isoperistaltic (ISO) or antiperistaltic (ANTI), with the choice largely based on the surgeon’s experience. This study aimed to evaluate these configurations regarding postoperative complications and operative outcomes. We searched PubMed, Scopus, and the Cochrane Central Register of Clinical Trials for studies published up to January 2025. Odds ratios (ORs) and mean differences (MDs), with 95% confidence intervals (CIs), were pooled using a random-effects model. Heterogeneity was assessed using the I² statistic, and analyses were conducted with R Software version 4.4.1.

Twelve studies involving patients undergoing colorectal surgery were included, comparing ISO and ANTI ileocolic anastomoses. ISO was associated with a significantly earlier return of flatus (MD: -0.3 days; 95% CI: -0.6 to -0.1; p<0.01). No statistically significant differences were found in anastomotic leak (OR: 0.61; 95% CI: 0.29-1.28; p=0.189), postoperative ileus (OR: 1.47; 95% CI: 0.87-2.50; p=0.149), anastomotic bleeding (OR: 0.70; 95% CI: 0.20-2.49; p=0.582), surgical site infection (SSI) (OR: 0.91; 95% CI: 0.38-2.17; p=0.829), reoperation (OR: 0.92; 95% CI: 0.47-1.82; p=0.813), time to first stool (MD: -0.3 days; 95% CI: -0.7 to 0.1; p=0.19), anastomotic time (MD: -0.2 minutes; 95% CI: -1.9 to 1.4; p=0.79), blood loss (MD: -4.0 mL; 95% CI: -17.8 to 9.8; p=0.57), operative time (MD: 4.2 minutes; 95% CI: -3.0 to 11.3; p=0.25), hospital stay (MD: -0.7 days; 95% CI: -1.7 to 0.4; p=0.19), or 30-day mortality (OR: 0.85; 95% CI: 0.25-2.86; p=0.787). Based on our findings, ISO and ANTI ileocolic anastomoses demonstrated comparable postoperative complication rates and operative outcomes. However, ISO was associated with a faster return of bowel function, evidenced by earlier passage of flatus.

## Introduction and background

Minimally invasive surgery, particularly the laparoscopic approach, is the predominant technique employed for colon resection [1-4]. After a right colectomy, ileocolic anastomoses can be performed using either side-to-side isoperistaltic (ISO) or antiperistaltic (ANTI) configuration, with the choice primarily depending on the surgeon’s expertise and preference [5,6]. Both methods are utilized in both open and laparoscopic procedures [1,2,5-7]. However, the impact of anastomotic configuration on short- and long-term outcomes remains insufficiently explored [4,5].

The ISO anastomotic approach is frequently favored due to its alignment with natural bowel peristalsis, which may contribute to reduced anastomotic tension [1,4,5]. Additionally, it typically requires less extensive bowel mobilization, potentially streamlining the surgical procedure and decreasing operative duration [5,6]. In contrast, the ANTI configuration creates a pseudo-valvular mechanism that may help regulate bowel transit, potentially reducing the risk of chronic diarrhea. However, this complication is rare in healthy adults without underlying gastrointestinal conditions such as irritable bowel syndrome or inflammatory bowel disease [4-6]. Additionally, this anastomotic orientation may decrease the likelihood of mesenteric twisting and has been suggested to lower the incidence of postoperative ileus compared to the isoperistaltic approach [1,4].

Several meta-analyses have compared intracorporeal and extracorporeal anastomoses without evaluating the type of anastomotic configuration [8,9]. However, no meta-analysis so far has specifically evaluated ISO versus ANTI approaches. Recently, randomized controlled trials (RCTs) have explored the impact of ileocolic anastomosis design after right colectomy [1,7]. In light of this, our objective is to evaluate ISO vs. ANTI configurations in right colectomies, with an emphasis on postoperative complications and surgical outcomes.

## Review

Materials and methods

This systematic review adhered to the Preferred Reporting Items for Systematic Reviews and Meta-Analyses (PRISMA) guidelines [10]. The study protocol was registered in the International Prospective Register of Systematic Reviews (PROSPERO) under the registration number CRD42024628834 [11]. Since this research is a systematic review and meta-analysis of previously published data, it does not require ethical approval. To ensure transparency and adherence to publication ethics, all included studies are properly cited, and standardized descriptions are used in the methodology where necessary. This approach follows the guidelines of the Committee on Publication Ethics (COPE) regarding text recycling, ensuring that any overlap is justified for clarity and reproducibility [12].

Search Strategy

A comprehensive search was conducted on PubMed, the Cochrane Central Register of Clinical Trials, and Scopus for studies published up to January 2025. The search strategy was structured as follows: (((isoperistaltic OR "end-to-end" OR end to end OR "side-to-side" OR “end-to-side” OR "end to end" OR "side to side" OR “end to side” OR triangulating OR triangular AND (antiperistaltic OR Barcelona)) OR “intracorporeal anastomosis” OR “extracorporeal anastomosis” OR “anastomotic type” OR “anastomotic configuration” OR “anastomotic method”) AND (colorectal OR ileocolic OR hemicolectomy OR colectomy OR “colon cancer”) AND (anastomosis OR anastomotic)) AND ((configuration OR type OR functional OR method) AND (anastomosis OR anastomotic)).

Eligibility Criteria

We included observational and RCTs that compared ISO and ANTI anastomoses, regardless of the surgical approach used (intracorporeal or extracorporeal) or the nature of the disease (benign or malignant). The exclusion criteria were as follows: (1) studies that solely assessed extracorporeal versus intracorporeal anastomotic techniques; (2) multiple anastomotic configurations performed; (3) studies without a control group; (4) publications deemed ineligible for inclusion, such as single-arm studies, case reports, conference abstracts, meta-analyses, reviews, and animal studies; and (5) studies with overlapping patient populations.

Data Extraction and Endpoints

Two researchers (B.F.P. and J.H.B.) independently reviewed the articles to determine eligibility and extracted data from the selected studies. Any disagreements were settled by mutual agreement or, when necessary, with input from a third reviewer (F.B.F.). The evaluated outcomes included postoperative complications, such as (1) anastomotic leakage, (2) ileus, (3) anastomotic bleeding, (4) time to first stool, (5) time to first flatus, (6) surgical site infection (SSI), (7) reoperation, (8) anastomotic time, (9) blood loss, (10) operative time, (11) length of hospital stay, and (12) 30-day mortality.

Quality Assessment

Two reviewers (B.F.P. and J.H.B.) independently evaluated the quality of the included studies using the Revised Cochrane Risk of Bias Tool (RoB 2) for RCTs [13]. This assessment classified each study into one of three categories: low risk, high risk, or some concerns, based on five key domains: randomization process, deviations from intended intervention, incomplete outcome data, outcome measurement, and selection of reported results. Similarly, the observational studies were evaluated using the Cochrane Collaboration tool to assess the risk of bias in non-randomized studies (ROBINS-I) [14]. In this assessment, each study was categorized as critical, serious, moderate, or low risk in the seven domains: confounding, selection, classification, deviations from intended interventions, missing data, measurement of outcomes, and selection of reported results. Any conflicts in the assessment were resolved through consensus with the senior author (F.B.F.).

Statistical Analysis

We calculated odds ratios (ORs) for categorical outcomes and mean differences (MDs) for continuous variables, both presented with 95% confidence intervals (CIs). A random-effects model was applied to all analyses. Statistical significance was set at p<0.05. Heterogeneity was evaluated using the Cochran Q test and the I² statistic, with p-values below 0.10 and I² values greater than 25% considered indicative of significant heterogeneity. For outcomes with high heterogeneity, Baujat plots were used to determine the influence of each study on the overall effect and variability. Additionally, leave-one-out sensitivity analyses were conducted by sequentially excluding each study from the pooled estimates to assess result stability. Publication bias was assessed through visual inspection of funnel plots and formally tested using Egger’s linear regression method for outcomes reported by at least 10 studies. Statistical analyses were performed using R Software (R Foundation for Statistical Computing), version 4.4.1.

Results

Selection and Characteristics of Included Studies

**Figure 1:**
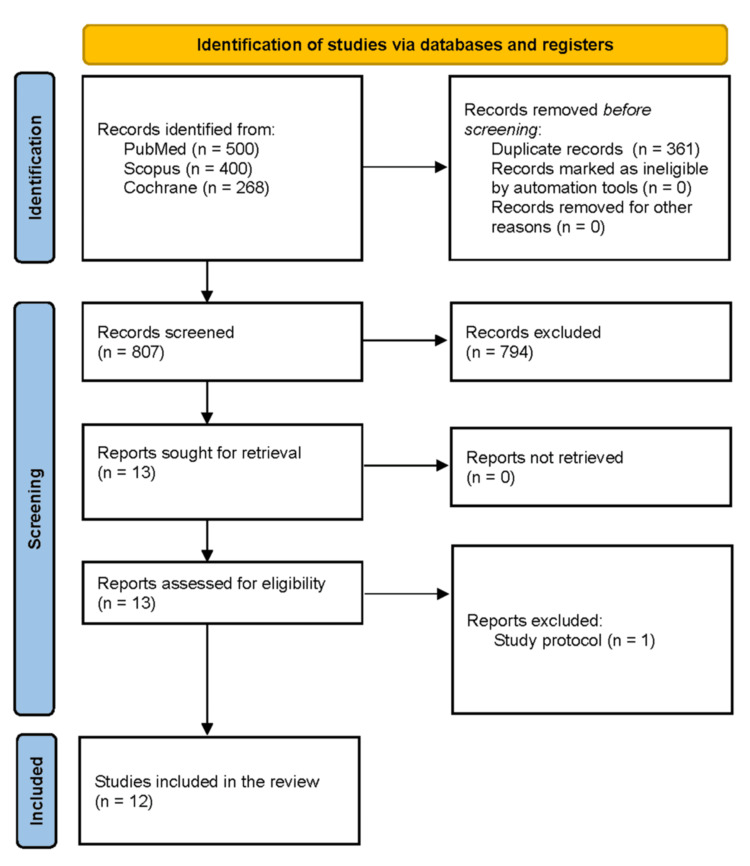
PRISMA flow diagram depicting study screening and selection PRISMA: Preferred Reporting Items for Systematic Reviews and Meta-Analyses

As shown in Figure 1, the initial search retrieved 1,168 studies. After removing 361 duplicates and excluding 794 articles based on title and abstract screening, a total of 12 studies, including two RCTs and 10 observational studies, were selected for final analysis [1,5,7,15-23]. One study was excluded at this stage because it was a research protocol, not a completed study, explaining the final inclusion of 12 studies instead of 13.

Altogether, these studies included 1,122 patients who underwent colorectal surgery, with 515 (45.9%) in the ISO group and 607 (54.1%) in the ANTI group [1,5,7,15-23]. Males accounted for 595 (52.5%) of the population [1,5,7,15-23]. The mean age was 63.2 ± 10.2 years in the ISO group and 63.0 ± 9.4 years in the ANTI group, while the mean BMI was 25.6 ± 2.5 kg/m² and 24.8 ± 2.2 kg/m², respectively [1,5,7,15-23]. Most patients were classified as American Society of Anesthesiologists (ASA) class I-II (59.2% ISO vs. 57.4% ANTI) and ASA III-IV (40.8% vs. 42.6%) [1,5,7,15-23]. Laparoscopic procedures were the predominant approach, performed in 94% of cases, whereas open surgeries accounted for 6% [1,5,7,15-23]. Six studies focused exclusively on patients with colon cancer, while the remaining included mixed populations with both benign and malignant conditions [1,5,7,15-23]. Only one study was limited to patients with inflammatory bowel disease. Regarding the anastomotic technique, six studies reported intracorporeal side-to-side ISO anastomoses, while ANTI configurations were commonly associated with extracorporeal approaches. Four studies exclusively involved intracorporeal anastomoses, and two reported only extracorporeal anastomoses. In all studies, stapling devices were used to construct anastomoses.

To date, only two RCTs have directly compared ISO and ANTI configurations. In the ISOVANTI trial, both groups underwent intracorporeal anastomosis, with laparoscopic suturing used for the closure of the enterotomies [1]. In contrast, Matsuda et al. employed linear staplers in the ISO group, creating a proximal ileal enterotomy on the antimesenteric border about 5 cm from the first staple line, while the colonic enterotomy was placed directly at the stapling edge [7]. Manual suturing was used for closure [7]. In the ANTI group, linear staplers were used throughout the anastomotic construction [7]. The trial was terminated early due to a higher-than-expected morbidity rate in the ISO group. Although not statistically significant, the trend raised concerns that led to the discontinuation of the trial [7]. Additional study characteristics are summarized in Tables 1-2.* *

**Table 1:** Baseline characteristics of the included studies ^a^No SD or range was given ANTI: antiperistaltic anastomosis; ISO: isoperistaltic anastomosis; NA: not available; RCT: randomized control trial

Study	Country	ISO/ANTI	Design	Sex, male, n (%); ISO/ANTI	BMI, kg/m², mean ± SD; ISO/ANTI	Age (years), mean ± SD; ISO/ANTI	ASA, n (%); ISO/ANTI	Disease	Follow-up, months, median (range
Chen et al. 2022 [15]	China	30/45	P-Obs	17 (56)/26 (57.7)	NA	33 ± 7.1/37 ± 9.7	NA	Crohn's disease	ISO: 21 (6–52)^**^;ANTI: 25 (6–49)
Chen 2023 [16]	China	58/56	R-Obs	26 (44.8)/30 (53.6)	23.9 ± 3.5/22.3 ± 2.8	61 ± 11/62 ± 11	NA	Benign: 8 (13.8)/10 (17.9); malignant: 50 (86.2)/46 (82.1)	1^a^
Erguner et al. 2013 [17]	Turkey	15/15	P-Obs	8 (53.4)/7 (46.6)	27 ± 3.4/25.8 ± 3.1	66.1 ± 9.4/63.1 ± 12.9	NA	Malignant: colon cancer	28 (5 - 99)
Fujii et al. 2024 [18]	Japan	17/17	R-Obs	9 (52.9)/12 (70.6)	23.5 ± 1.1/23.7 ± 1.6	71.8 ± 2.8/72.6 ± 3.3	I - II: 15 (88.2)/14 (82.4); III-IV: 2 (11.8)/(17.6)	Malignant: colon cancer	6^a^
Gil et al. 2017 [19]	Portugal	31/84	R-Obs	17 (54.8)/46 (54.7)	NA	72 ± 12.8/69 ± 13.2	I - II: 15 (48.3)/52 (61.9); III-IV: 16 (51.7)/32 (38.1)	Benign: 1 (3.2)/2 (2.4); malignant: 30 (96.8)/82 (97.6)	One^a^
Hanna et al. 2016 [20]	USA	86/109	R-Obs	41 (47.67)/46 (42.20)	25.9 ± 1.3/25.1 ± 1.6	65.8 ± 4.9/58.9 ± 5.3	I: 0 (0)/0 (0); II: 36 (41.8)/50 (45.8); III: 42 (48.8)/53 (41.8); IV: 8 (9.3)/6 (5.50)	Benign: 30 (34.8)/52 (47.7); malignant: 56 (65.2)/57 (52.3)	ISO: 7.3 ( 1.9 - 24.7); ANTI: 10.1 (2.2 - 26.9)
Hellan et al. 2009 [21]	USA	23/57	R-Obs	16 (69.5)/29 (50.8)	27.9 ± 5.4/28.1 ± 4.3	67.2 ± 9.0/66.8 ± 12.2	NA	Benign: 6 (26)/19 (33.4); malignant 17 (74)/38 (66.6)	1^a^
ISOVANTI 2018 [1]	Spain	54/54	RCT	36 (66.6)/33 (61.1)	27.3 ± 1.0/27.2 ± 1.6	68.2 ± 10.8/68.8 ± 10.3	I: 1 (1.9)/4 (7.4); II: 30 (55.5)/25 (46.2); III: 20 (37.0)/23 (42.5); IV: 3 (5.5)/2 (3.7)	Malignant: colon cancer	1 (short-term)^a^; 12 (long-term)
22]	Japan	15/17	R-Obs	7 (46.6)/10 (58.8)	22.2 ± 4.5/22.5 ± 4.1	67.1 ± 10.06/71 ± 8.9	I: 11 (73.4)/14 (82.3); II: 3 (20)/3 (17.7); III: 1 (6.6)/0 (0)	Malignant: colon cancer	1^a^
Kwiatkowski et al. 2019 [23]	Poland	51/34	R-Obs	31 (60.8)/22 (64.7)	28.1 ± 4.1/28.1 ± 5.5	65.2 ± 11.5/65.9 ± 13.6	I: 5 (9.8)/3 (9.1); II: 33 (64.7)/22 (66.7); III: 13 (25.5)/7 (8.3); IV: 0 (0)/1 (1.2)	Benign: 8 (15.7)/6 (17.6); malignant 43 (84.3)/28 (82.4)	1^a^
Matsuda et al. 2015 [7]	Japan	20/20	RCT	11 (55)/11 (55)	22.6 ± 2.8/23.3 ± 3.5	66 ± 12/68 ± 10	I: 2 (10)/6 (30); II: 18 (90)/13 (75); III: 0 (0)/1 (5)	Malignant: colon cancer	1^a^ (discontinuation of the study)
Zhang et al. 2022 [5]	China	115/99	R-Obs	61 (53.0)/43 (43.4)	24.1 ± 3.3/24.0 ± 3.6	60.4 ± 11.4/59.4 ± 12.2	NA	Malignant: colon cancer	ISO: 35.6^a^; ANTI: 35.3

**Table 2:** Clinical and treatment characteristics of the included studies ^a^A stapler used in both anastomosis configurations ANTI: antiperistaltic anastomosis; ISO: isoperistaltic anastomosis; NA: not available or not applicable

Author	Surgical Approach	Anastomosis Approach	Intracorporeal	Extracorporeal	Stapler used	Suture for Enterotomy in Iso	Specimen Extraction	Mesenteric defect
	N (%)				(Iso/Anti)			Closure
Chen et al. 2022 [15]	Open: 67 (90)	Extra	NA	ISO + ANTI	NTLC75 (Ethicon)^a^	3-0 Vicryl	Small incision (not specified)	Closed if feasible
	Laparoscopic: 8 (10)							
Chen et al. 2023 [16]	Laparoscopic (100)	Intra + extra	ISO	ANTI	Linear stapler^a^	3-0 Vicryl	Paraumbilical incision	NA
Erguner et al. 2013 [17]	Laparoscopic (100)	Intra + extra	ISO	ANTI	Echelon 60 ENDOPATH (Ethicon)^a^	2-0 Polypropylene	Pfannenstiel	Not closed
Fujii et al. 2024 [18]	Laparoscopic (100)	Extra	NA	ISO + ANTI	60-mm Linear stapler^a^	Not reported	Midline incision (umbilical extension)	NA
Gil et al. 2017 [19]	Laparoscopic (100)	Intra + extra	ISO	ANTI	60-mm endostapler/80-mm stapler	Mid-term absorbable braided suture	Median mini-laparotomy or paraumbilical incision	NA
Hanna et al. 2016 [20]	Laparoscopic (100)	Intra + extra	ISO	ANTI	60-mm linear stapler/75-mm stapler	3-0 Vicryl	Midline incision (umbilical extension), Pfannenstiel transvaginal	Not closed
Hellan et al. 2009 [21]	Laparoscopic (100)	Intra + extra	ISO	ANTI	Endo-GIA^a^	3-0 Vicryl	Midline incision (umbilical extension)	Not closed
ISOVANTI 2018 [1]	Laparoscopic (100)	Intra	ISO + ANTI	NA	Endo-GIA^a^	Continuous suture	Pfannenstiel (or prior laparotomy site)	NA
Ishizak et al. 2018 [22]	Laparoscopic (100)	Intra	ISO + ANTI	NA	Endo-GIA ^a^	Two-layer hand-sewn suture	Pfannenstiel	Closed
Kwiatkowski et al. 2019 [23]	Laparoscopic (100)	Intra + extra	ISO	ANTI	60-mm endostapler/55-mm GIA	Double-layer continuous suture	Pfannenstiel	Not closed
Matsuda et al. 2015 [7]	Laparoscopic (100)	Intra	ISO + ANTI	NA	Echelon 60-mm (Ethicon)^a^	4-0 PDS II	NA	NA
Zhang et al. 2022 [5]	Laparoscopic (100)	Intra	ISO + ANTI	NA	Endoscopic linear stapler^a^	Single-layer continuous suture	Pfannenstiel	NA

Pooled Analyses of the Included Studies

**Figure 2:**
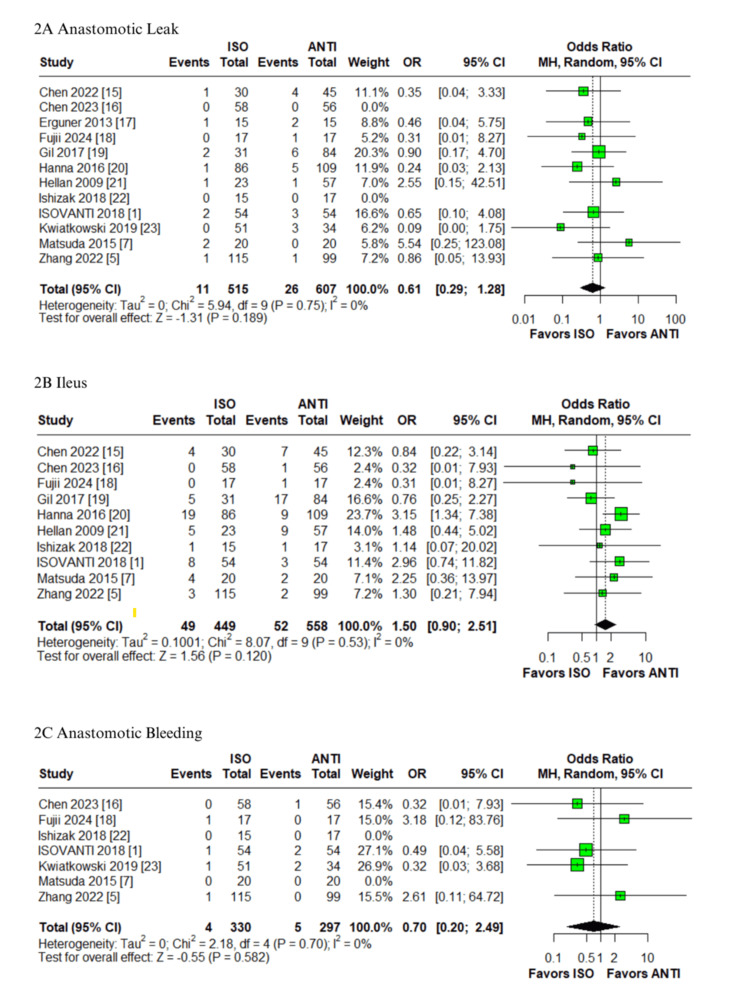
Forest plots comparing isoperistaltic and antiperistaltic anastomoses in right colectomies: (A) anastomotic leak; (B) ileus; (C) anastomotic bleeding ANTI: antiperistaltic anastomosis; CI: confidence interval; ISO: isoperistaltic anastomosis; IV; inverse variance; MH: Mantel-Haenszel; OR: odds ratio

**Figure 3:**
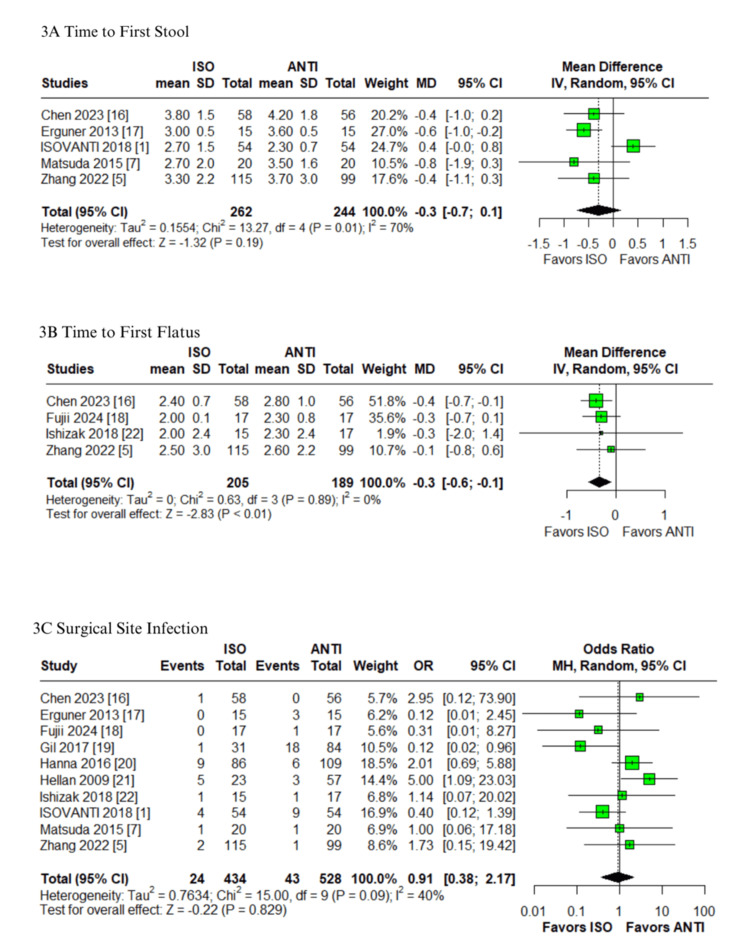
Forest plots comparing isoperistaltic and antiperistaltic anastomoses in right colectomies: (A) first stool; (B) first flatus; (C) SSI ANTI: antiperistaltic anastomosis; CI: confidence interval; ISO: isoperistaltic anastomosis; IV; inverse variance; MD: mean difference; MH: Mantel-Haenszel; OR: odds ratio; SD: standard deviation; SSI: surgical site infection

**Figure 4:**
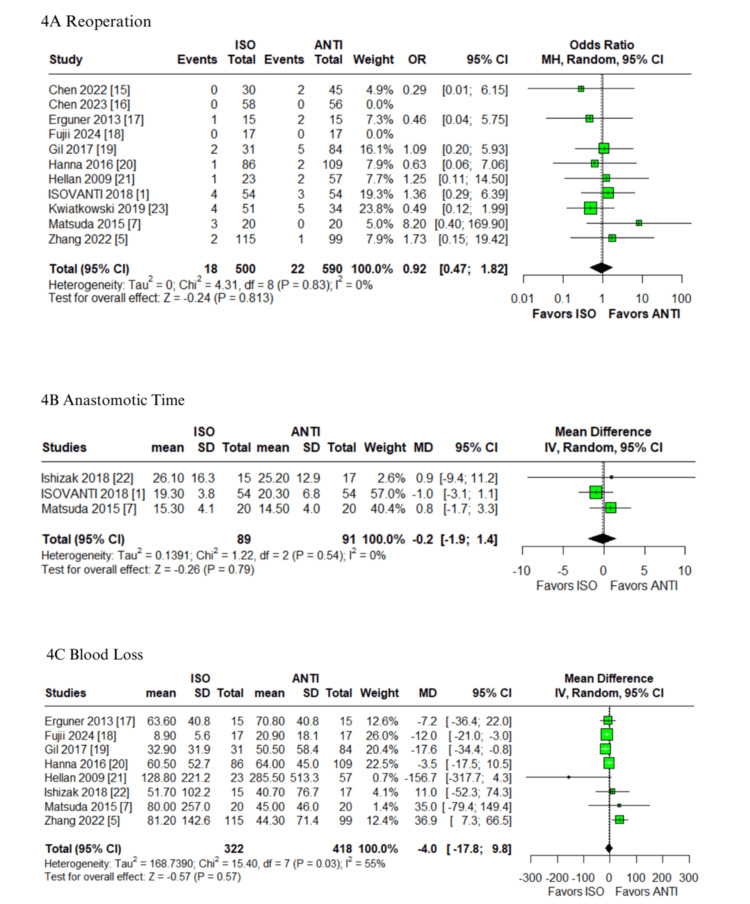
Forest plots comparing isoperistaltic and antiperistaltic anastomoses in right colectomies: (A) reoperations; (B) anastomotic time; (C) blood loss ANTI: antiperistaltic anastomosis; CI: confidence interval; ISO: isoperistaltic anastomosis; IV; inverse variance; MD: mean difference; MH: Mantel-Haenszel; OR: odds ratio; SD: standard deviation

Postoperative complications: In the pooled analysis of patients undergoing colorectal surgery with ISO versus ANTI anastomoses, no statistically significant differences were observed in the anastomotic leak (OR: 0.61; 95% CI: 0.29-1.28; p=0.189; I²=0%; Figure 2A) [1,5,7,15-23], postoperative ileus (OR: 1.47; 95% CI: 0.87-2.50; p=0.149; I²=0%; Figure 2B) [1,5,7,15,16,18-22], anastomotic bleeding (OR: 0.70; 95% CI: 0.20-2.49; p=0.582; I²=0%; Figure 2C) [1,5,7,16,18,22,23], or time to first stool (MD: -0.3 days; 95% CI: -0.7 to 0.1; p=0.19; I²=70%; Figure 3A) [1,5,7,16,17]. The first three outcomes showed low heterogeneity, while the latter demonstrated high heterogeneity. The ISO group was associated with a significantly earlier return of flatus compared to the ANTI group (MD: -0.3 days; 95% CI: -0.6 to -0.1; p<0.01; I²=0%; Figure 3B) [5,16,18,22], with low heterogeneity. Similarly, no significant differences were identified in the rates of SSI (OR: 0.91; 95% CI: 0.38-2.17; p=0.829; I²=40%; Figure 3C) or reoperation (OR: 0.92; 95% CI: 0.47-1.82; p=0.813; I²=0%; Figure 4A) [1,5,7,15-23]. All remaining outcomes exhibited low heterogeneity.

**Figure 5:**
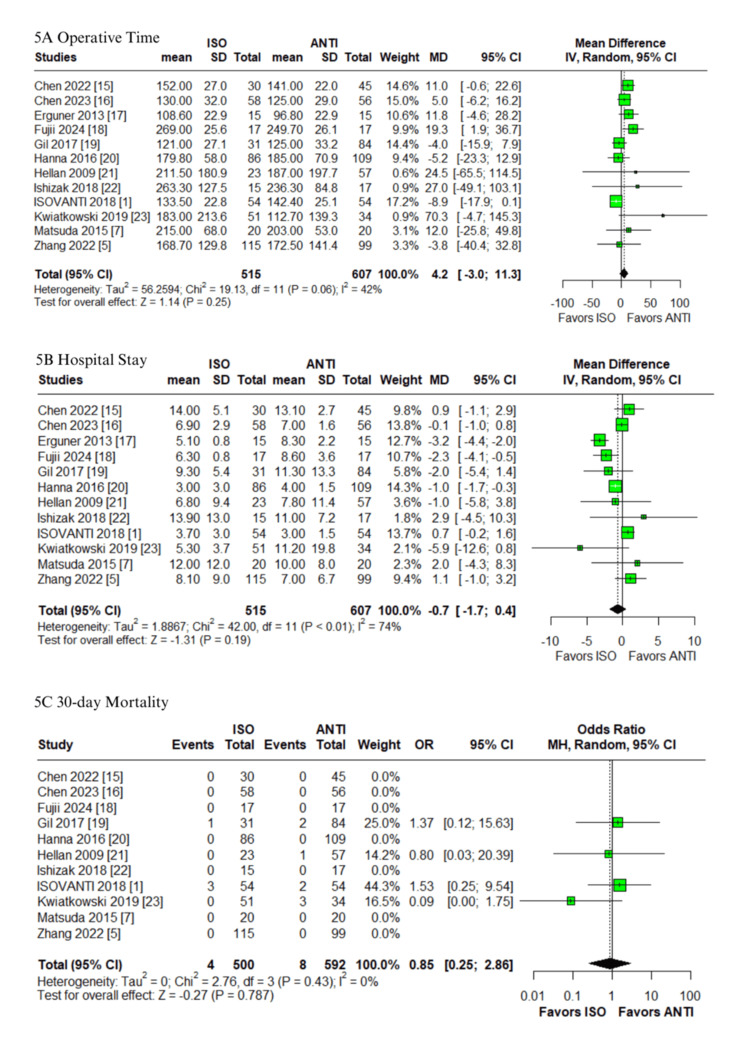
Forest plots comparing isoperistaltic and antiperistaltic anastomoses in right colectomies: (A) operative time; (B) hospital stay; (C) 30-day mortality ANTI: antiperistaltic anastomosis; CI: confidence interval; ISO: isoperistaltic anastomosis; IV; inverse variance; MD: mean difference; MH: Mantel-Haenszel; OR: odds ratio; SD: standard deviation

Operative outcomes and mortality​​​: No significant differences were found between groups in anastomotic time (MD: -0.2 minutes; 95% CI: -1.9 to 1.4; p=0.79; I²=0%; Figure 4B) [1,7,22], blood loss (MD: -4.0 mL; 95% CI: -17.8 to 9.8; p=0.57; I²=55%; Figure 4C) [5,7,17-22], operative time (MD: 4.2 minutes; 95% CI: -3.0 to 11.3; p=0.25; I²=42%; Figure 5A) [1,5,7,15-23], hospital stay (MD: -0.7 days; 95% CI: -1.7 to 0.4; p=0.19; I²=74%; Figure 5B) or 30-day mortality (OR: 0.85; 95% CI: 0.25-2.86; p=0.787; I²=0%; Figure 5C) [1,5,7,15-23]. Anastomotic time, 30-day mortality, and operative time were associated with low and moderate heterogeneity, respectively. In contrast, both blood loss and hospital stay demonstrated high heterogeneity.

Quality Assessment

**Figure 6:**
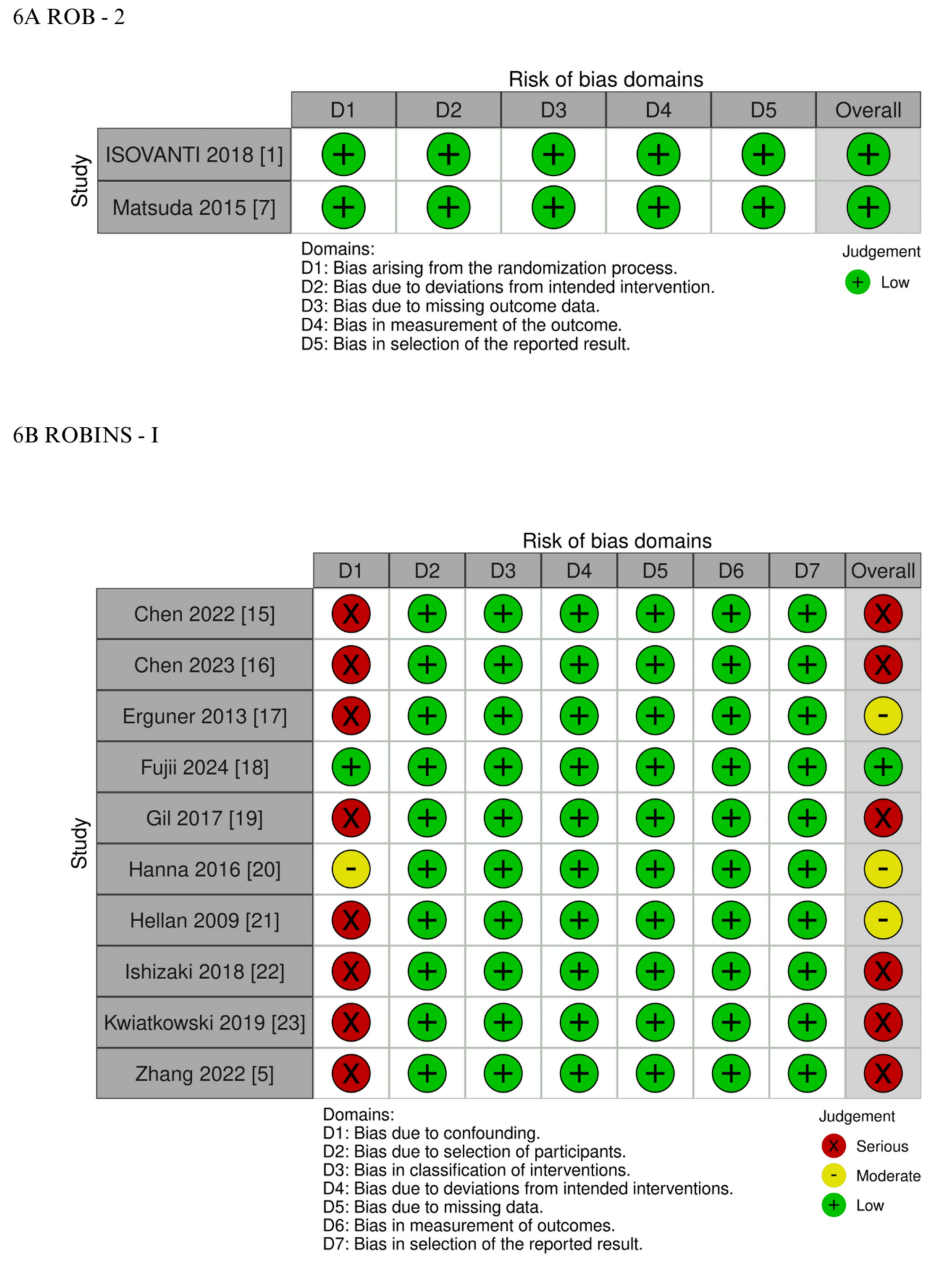
Critical appraisal of non-randomized and randomized controlled trials according to the Cochrane Collaboration’s tool for assessing risk of bias: (A) Rob 2; (B) ROBINS-I RoB 2: Revised Cochrane Risk of Bias Tool; ROBINS-I: Risk of Bias In Non-randomized Studies – of Interventions

The individual risk-of-bias assessment for each study included in the meta-analysis is presented in Figure 6. The two RCTs were evaluated using the RoB 2 tool and were both classified as having a low risk of bias [1,7,13]. Among the 10 observational studies, one was judged to have a low risk of bias after applying the propensity score matching methodology, and another was considered to have a moderate risk of bias based on multivariable adjustment of the primary outcomes [18,20]. The remaining eight studies were classified as having a serious risk of bias [5,15-17,19,21-23]. The main contributor to bias in the observational studies was confounding, as reflected in the “bias due to confounding” domain of the ROBINS-I tool, which significantly influenced the overall risk assessment [14].

Sensitivity Analyses

**Figure 7:**
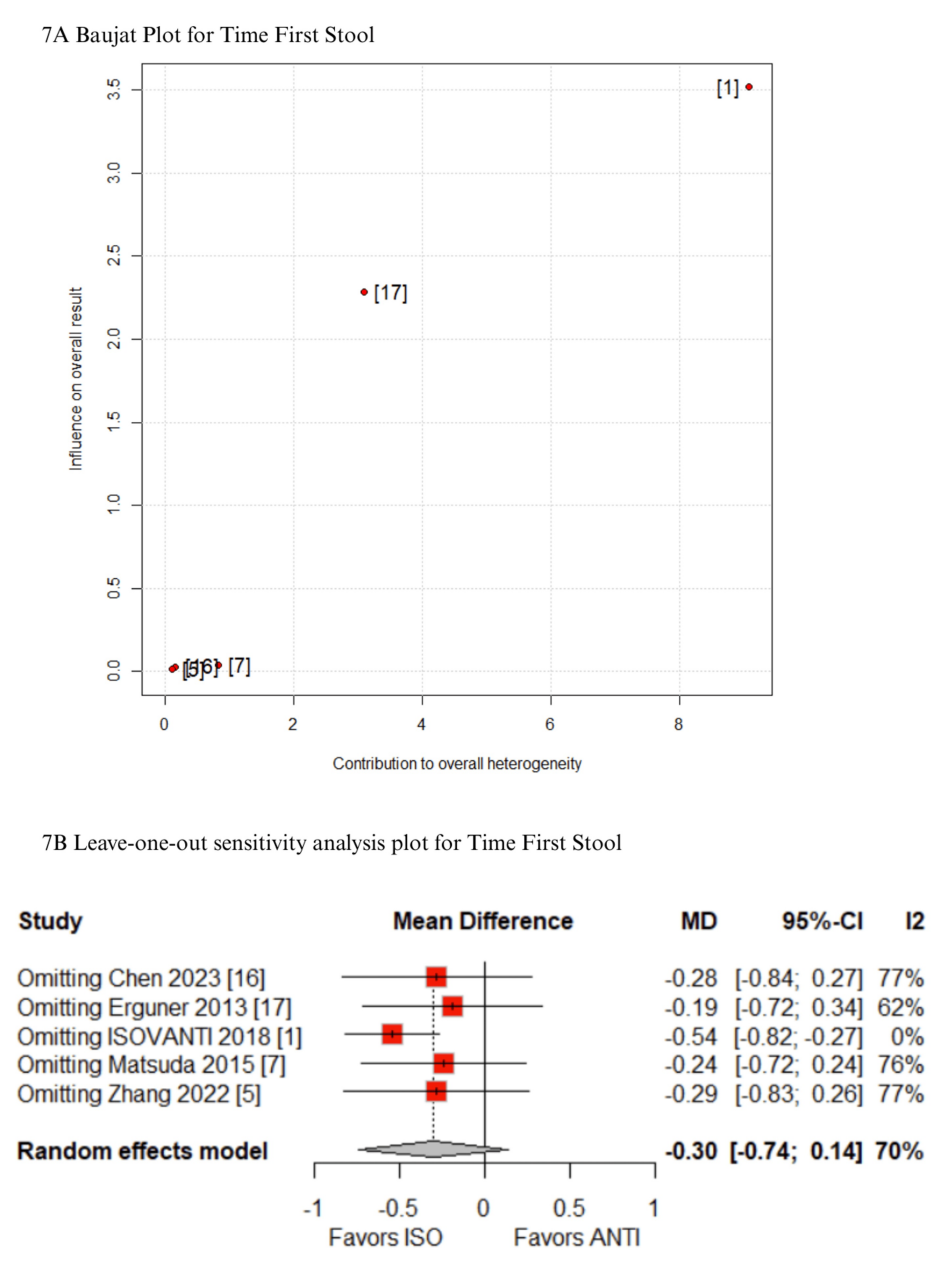
Sensitivity analyses of time to first stool ANTI: antiperistaltic anastomosis; CI: confidence interval; ISO: isoperistaltic anastomosis; MD: mean difference

**Figure 8:**
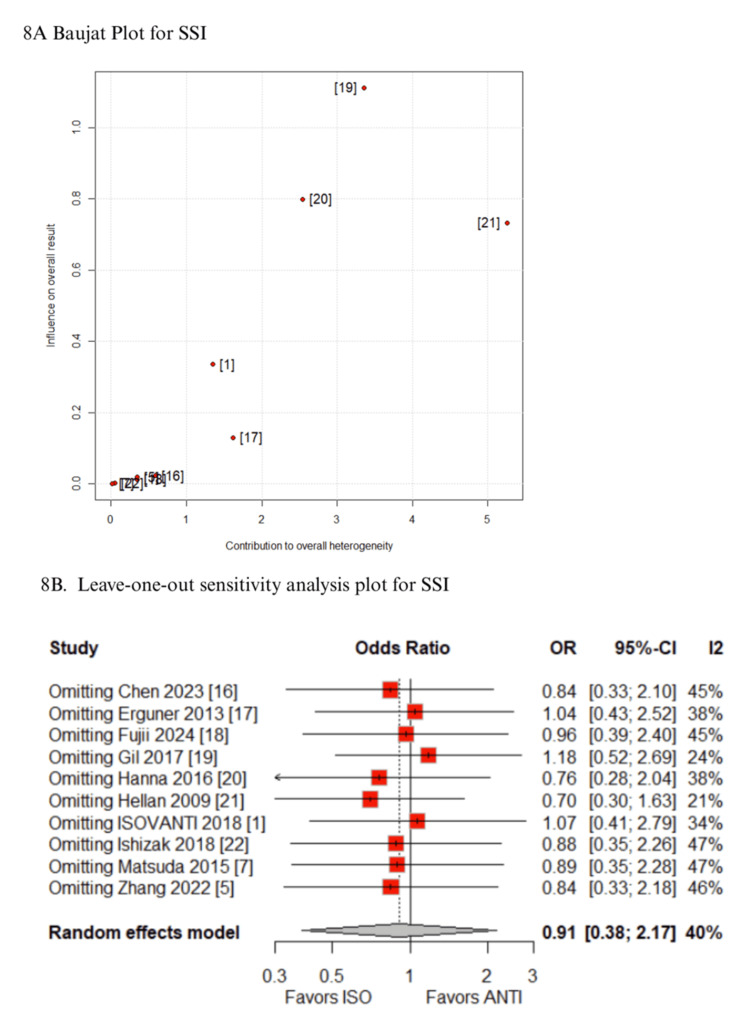
Sensitivity analyses of SSI ANTI: antiperistaltic anastomosis; CI: confidence interval; ISO: isoperistaltic anastomosis; OR: odds ratio; SSI: surgical site infection

**Figure 9:**
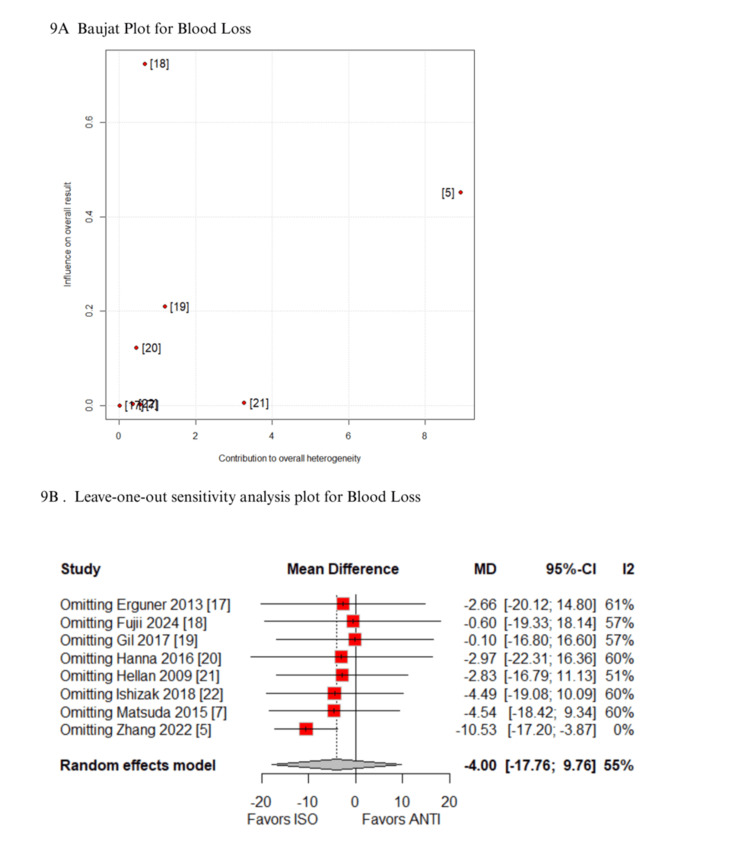
Sensitivity analysis of blood loss ANTI: antiperistaltic anastomosis; CI: confidence interval; ISO: isoperistaltic anastomosis; MD: mean difference

**Figure 10:**
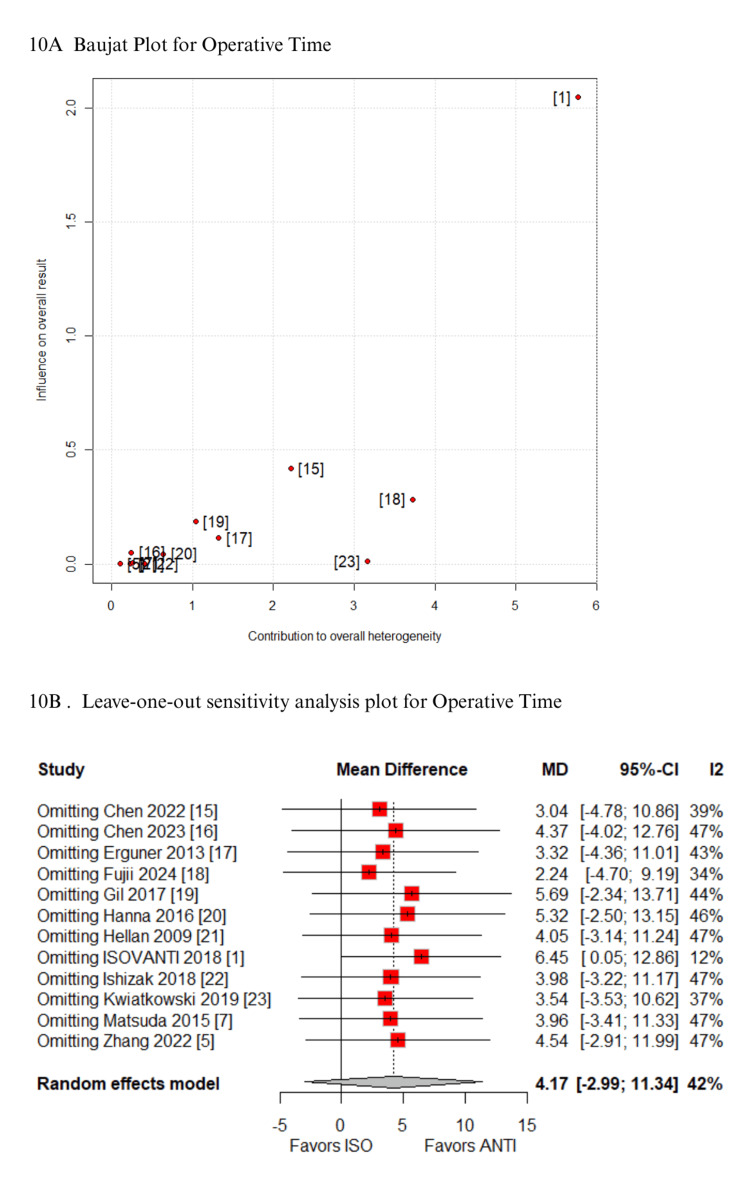
Sensitivity analysis of operative time ANTI: antiperistaltic anastomosis; CI: confidence interval; ISO: isoperistaltic anastomosis; MD: mean difference

**Figure 11:**
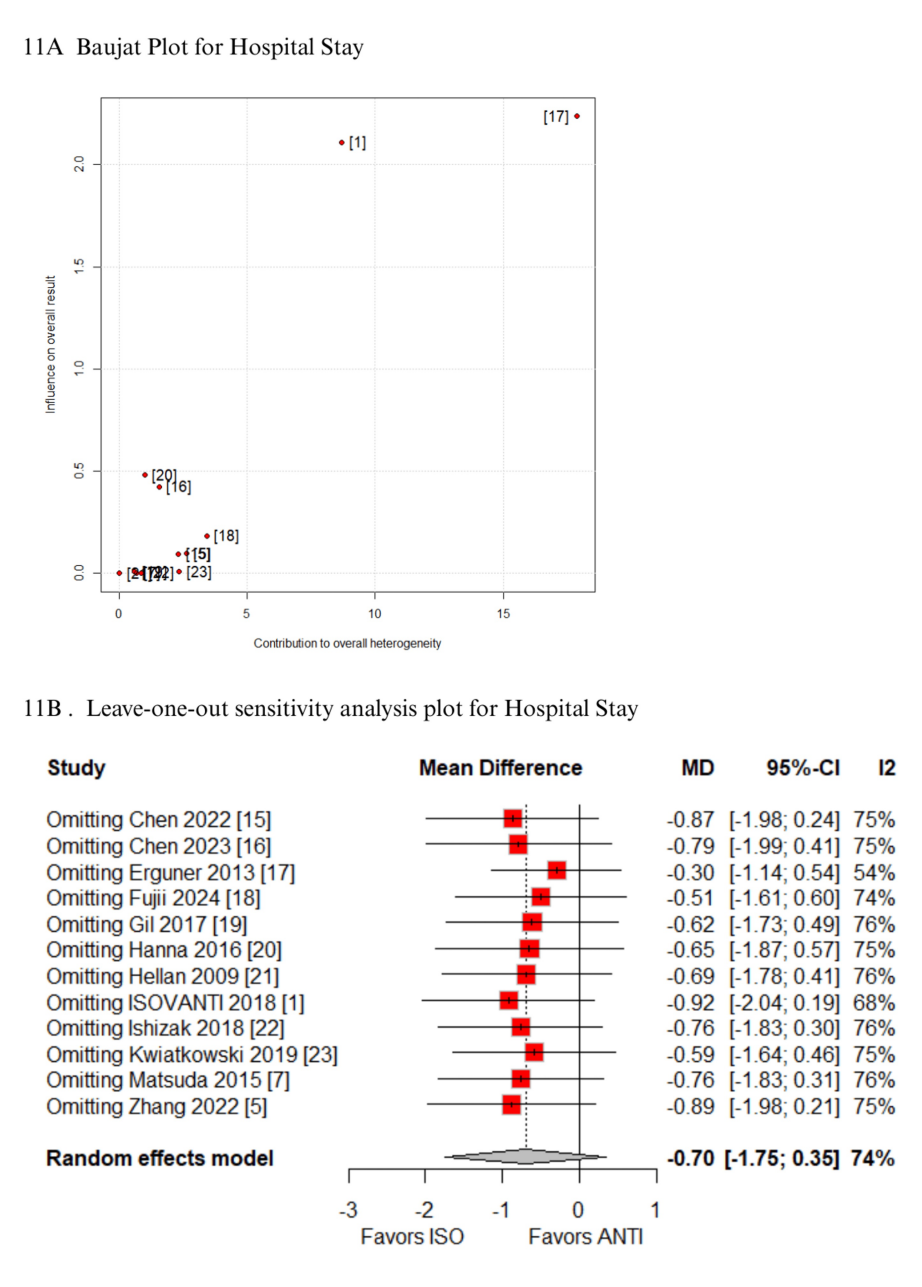
Sensitivity analysis of hospital stay ANTI: antiperistaltic anastomosis; CI: confidence interval; ISO: isoperistaltic anastomosis; MD: mean difference

In the Baujat plot analysis, the studies that contributed most to heterogeneity were identified. For the outcome of time to first stool, the ISOVANTI trial emerged as the main source of heterogeneity [1]. After its exclusion in the leave-one-out sensitivity analysis, the result became statistically significant in favor of the ISO group (MD: -0.54 days; 95% CI: -0.82 to -0.27) (Figures 7A-7B) [1]. Regarding SSI, the study by Hellan et al. contributed most to heterogeneity [21]. However, its exclusion did not alter the overall findings, which remained consistent (Figures 8A-8B) [21]. Similarly, for blood loss, Zhang et al. were identified as the major contributor to heterogeneity [5]. Yet, the exclusion of this study had no impact on the pooled result (Figures 9A-9B) [5]. When analyzing operative time, the ISOVANTI trial again stood out as the primary source of heterogeneity (Figures 10A-10B) [1]. The leave-one-out analysis confirmed that the results remained stable following its removal [1]. Finally, for the outcome of hospital stay, Erguner et al.' study was also identified as the main contributor to heterogeneity [1]. The exclusion of this study did not significantly affect the overall effect estimate (Figures 11A-11B) [1].

**Figure 12:**
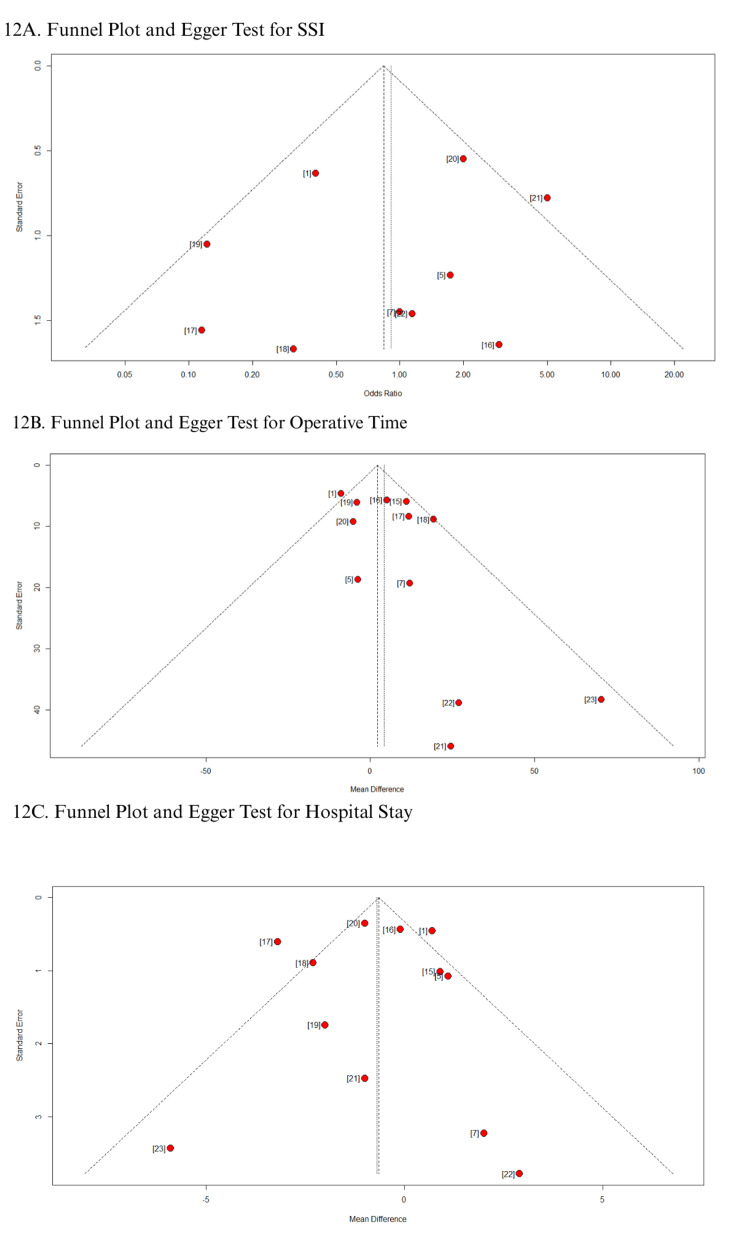
Funnel plot and Egger test: (A) SSI; (B) operative time; (C) hospital stay SSI: surgical site infection

**Figure 13:**
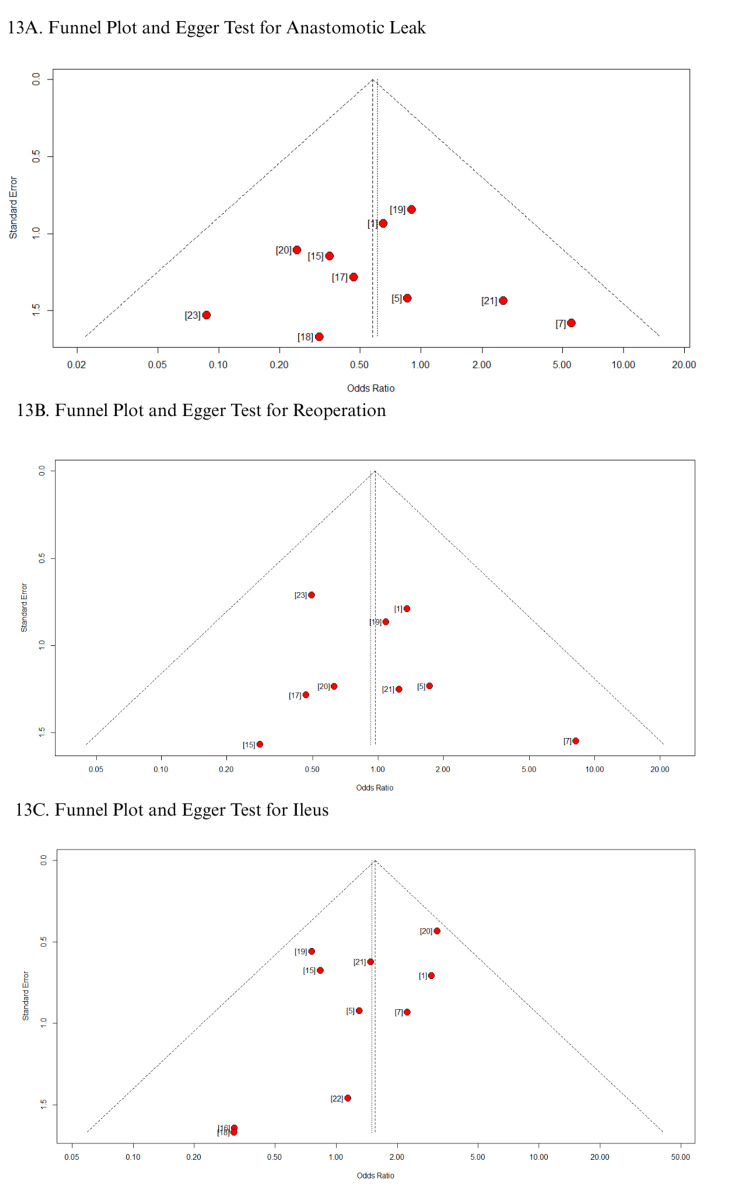
Funnel plot and Egger test: (A) anastomotic leak; (B) reoperation; (C) ileus

In addition, funnel plots for all secondary outcomes demonstrated a symmetrical distribution of studies around the pooled effect size, suggesting no visual evidence of publication bias (Figures 12-13) [1,5,7,15-23]. This observation was corroborated by Egger’s regression test, which showed no statistically significant small-study effects (as shown in Table 3). These findings consistently indicate the absence of publication bias across all evaluated outcomes.

**Table 3:** Egger test results

Outcome	No. of studies	Test result	Bias estimate
Surgical site infection	9	t = -0.76, df = 8, p-value = 0.4691	-0.7991 (SE = 1.0516)
Operative time	11	t = 1.78, df = 10, p-value = 0.1055	1.1707 (SE = 0.6578)
Hospital stay	11	t = -0.10, df = 10, p-value = 0.9220	-0.1002 (SE = 0.9978)
Anastomotic leak	9	t = 0.03, df = 8, p-value = 0.9749	0.0394 (SE = 1.2163)
Reoperation	8	t = 0.62, df = 7, p-value = 0.5525	0.5793 (SE = 0.9284)
Ileus	9	t = -1.69, df = 8, p-value = 0.1291	-1.1913 (SE = 0.7040)

Discussion

This systematic review and meta-analysis is the first to provide a comprehensive statistical synthesis comparing ISO and ANTI ileocolic anastomoses following right colectomy. Based on two RCTs and 10 observational studies, including a total of 1,122 patients undergoing colorectal surgery, our analysis demonstrated that the ISO configuration was associated with a significantly earlier return of flatus compared to the ANTI group. No statistically significant differences were observed between the two techniques regarding anastomotic leak, postoperative ileus, anastomotic bleeding, SSI, reoperation, time to first stool, anastomotic time, intraoperative blood loss, operative time, length of hospital stay, or 30-day mortality.

**Figure 14:**
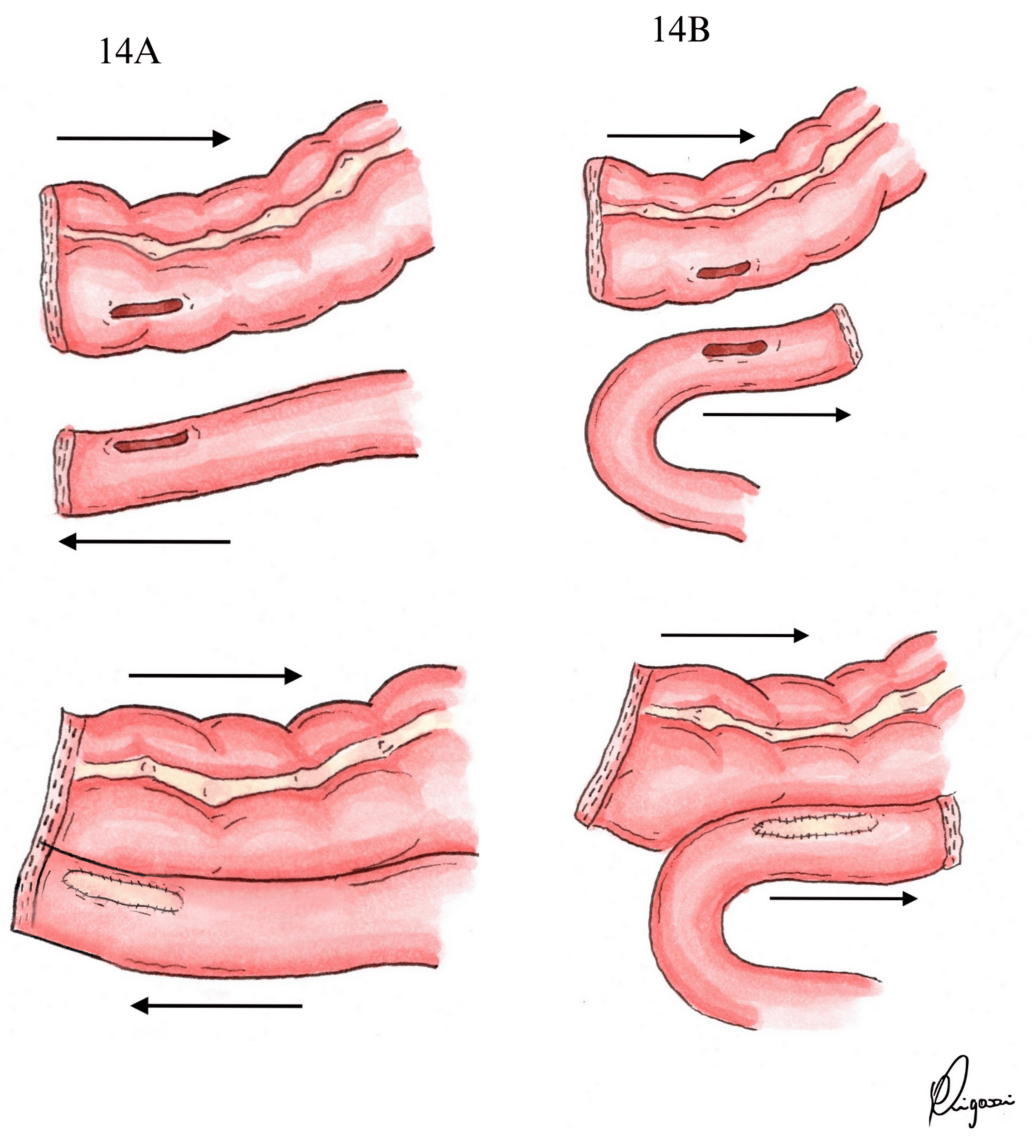
Surgical technique (A) Side-to-side antiperistaltic ileocolic anastomosis; (B) Side-to-side isoperistaltic ileocolic anastomosis Illustration by the author (Pigossi B)

The rise of minimally invasive surgery and natural orifice specimen extraction (NOSE) has reinforced the relevance of comparing intracorporeal and extracorporeal anastomotic techniques [8,9]. In the studies included in this review, ISO configurations were predominantly performed during intracorporeal anastomosis, whereas ANTI configurations were typically associated with the extracorporeal approach [1,5,7,15-23]. This distinction may reflect both anatomical considerations and technical preferences. In the extracorporeal approach, specimen extraction and anastomosis construction often occur through the same access point, which can offer logistical advantages in selected cases. Conversely, intracorporeal techniques offer greater flexibility in anastomotic orientation, albeit at the cost of requiring advanced laparoscopic skills, particularly in suturing. Figure 14 demonstrates the technical aspects of both anastomosis configurations.

ANTI, also referred to as functional end-to-end anastomosis (FEEA), was first described in 1968 and employs a linear stapler device [24]. This approach streamlines the procedure, decreasing intestinal leak, operative time, and minimizing dependence on the surgeon's technical skill compared to hand-sewn methods [7,24,25]. Additionally, ANTI reduces the likelihood of mesenteric twisting, which can be more common in ISO configurations. Its pseudo-valvular mechanism may also help mitigate the risk of chronic diarrhea [1,4,21]. On the other hand, ISO anastomosis follows the natural direction of bowel peristalsis, which may decrease anastomotic tension [5]. Moreover, it requires less bowel mobilization, which may simplify the procedure and reduce the incidence of ileus [1,4,5,7].

In terms of postoperative complications, including ileus, anastomotic bleeding, reoperation, and SSI, we observed overall low rates, with no statistically significant differences between ISO and ANTI groups. Similarly, Zhang et al. reported comparable complication rates for both intracorporeal side-to-side anastomoses [5]. In contrast, Baqar et al., comparing extracorporeal ANTI and end-to-side (ES) anastomoses, observed significantly higher rates of postoperative ileus in the ANTI group (p<0.001), highlighting the potential impact of anastomotic configuration and surgical approach on clinical outcomes [26]. Regarding ISO anastomoses, Wu et al. found no significant differences in postoperative complications when compared to other hand-sewn techniques [27]. In our analysis, ISO anastomoses were associated with a faster return of flatus and, after sensitivity analysis, earlier passage of stool compared to ANTI. These findings may be attributed to reduced bowel mobilization required in the isoperistaltic technique [1,4,5,7]. Another contributing factor could be the wider anastomotic lumen typically observed in ISO configurations when compared to ANTI, which may facilitate more efficient transit in the early postoperative period [27].

Focusing on anastomotic leakage, while rare (3%-5%), carries significant morbidity and mortality risks [28-30]. Kracht et al. demonstrated a lower leakage rate with stapled ANTI (2.8%) compared to other manual techniques (8.3%; p<0.02) [31]. Liu et al. reported similarly low leakage rates (0.5%-1.8%), underscoring the safety of both ANTI and end-to-side configurations [32]. For the isoperistaltic configuration compared to other anastomosis configurations, Wu et al. found no significant difference in rates of anastomotic leaks [27]. Additionally, recent trials analyzing intracorporeal (IA) and extracorporeal anastomoses (EA), both ISO, showed consistent findings. For instance, Vignali et al. reported a 6.6% leakage rate in IA and none in EA, whereas Ferrer-Márquez et al. observed an overall leakage rate of 6.25% with no group differences [33,34].

The duration of surgery serves as a key quality metric, as prolonged operative times can elevate the likelihood of postoperative complications [35]. Liu et al. reported shorter operative times with ANTI compared to end-to-side anastomoses (140.4 ± 14.9 min vs. 150.5 ± 20.1 min; p=0.001) [32]. Similarly, Baqar et al. observed faster procedures with ANTI compared to ES (p<0.001) [26]. In contrast, Zhang et al. found no significant differences in operative times between ISO and ANTI intracorporeal anastomoses, aligning with our findings of comparable times between the groups [5]. These findings suggest that while certain configurations may offer specific theoretical advantages, the outcomes of ileocolic anastomoses are more likely influenced by the surgeon's experience and institutional expertise than by the anastomotic configuration itself. This highlights the importance of proper training and proficiency in both techniques to ensure optimal patient outcomes.

This study has several limitations. First, although the analysis included 1,122 patients across 12 studies, only two were RCTs, and the remaining were observational in design. This may limit the strength of the evidence, especially for outcomes with low incidence, such as anastomotic leak or mortality. Second, despite focusing on ISO and ANTI configurations, the included studies demonstrated considerable heterogeneity in anastomotic technique, including variations in the use of linear staplers, hand-sewn methods, and intracorporeal versus extracorporeal approaches. Given the frequent association of ISO with intracorporeal techniques and ANTI with extracorporeal techniques, future studies should consider conducting stratified analyses based on anastomotic approach to better isolate the effects of configuration from those of surgical technique. Third, the patient populations were diverse, encompassing both benign conditions and malignant diseases, which differ in surgical complexity and risk of complications. Fourth, the analysis combined data from open and laparoscopic procedures, without uniform reporting or stratification by surgical access, which may have influenced the results. Finally, most procedures were performed in high-volume centers by experienced surgeons, limiting the generalizability of our findings to lower-volume institutions or less experienced teams. These limitations highlight the need for larger, high-quality, and standardized trials to better isolate the impact of anastomotic configuration on surgical outcomes.

## Conclusions

In this systematic review and meta-analysis, which included two RCTs and 10 observational studies involving 1,122 patients undergoing colorectal surgery, no significant differences were observed between ISO and ANTI anastomoses in terms of anastomotic leak, ileus, anastomotic bleeding, time to first stool, SSI, reoperation, anastomotic time, operative time, intraoperative blood loss, length of hospital stay, or 30-day mortality. However, ISO was associated with a significantly earlier return of flatus compared to ANTI, suggesting a potential functional benefit in postoperative bowel recovery.
